# Supplementary material for: Rapid metabolism fosters microbial survival in the deep, hot subseafloor biosphere
Source: Nat Commun. 2022 Jan 25;13:312. doi: 10.1038/s41467-021-27802-7 (PMC8789916; doi:10.1038/s41467-021-27802-7)
Supplement: Supplementary file 1 — Supplementary Information [file 41467_2021_27802_MOESM1_ESM.pdf]

RAPID METABOLISM FOSTERS MICROBIAL SURVIVAL IN THE DEEP, HOT, SUBSEAFLOOR BIOSPHERE

F. BEULIG[1], F. SCHUBERT[2], R.R. ADHIKARI[3], C. GLOMBITZA[4], V.B. HEUER[3], K.-U. HINRICHS[3], K.L. HOMOLA[5], F. INAGAKI[6,7], B.B. JØRGENSEN[1], J. KALLMEYER[2], S.J.E. KRAUSE[8], Y. MORONO[6], J. SAUVAGE[5,9], A.J. SPIVACK[5], T. TREUDE[8,10,*]

[1] Center for Geomicrobiology, Department of Bioscience, Aarhus University, Ny Munkegade 114, 8000 Aarhus C, Denmark

[2] GFZ German Research Center for Geosciences, Section 3.7 Geomicrobiology, Telegrafenberg, 14473 Potsdam, Germany

[3] MARUM-Center for Marine Environmental Sciences, University of Bremen, Leobener Strasse 8, 28359 Bremen, Germany

[4] Department of Environmental Systems Science, ETH Zürich, Universitätstrasse 16, 8092 Zürich, Switzerland

[5] Graduate School of Oceanography, University of Rhode Island, Narragansett Bay Campus, 215 South Ferry Road, RI 02882, USA

[6] Kochi Institute for Core Sample Research, Japan Agency for Marine-Earth Science and Technology (JAMSTEC), Nankoku, Kochi 783-8502, Japan

[7] Mantle Drilling Promotion Office, Institute for Marine-Earth Exploration and Engineering, JAMSTEC, Yokosuka 237-0061, Japan

[8] Department of Earth, Planetary and Space Sciences, University of California Los Angeles, Los Angeles, CA 90095, USA

[9] Current address: Department of Marine Sciences, University of Gothenburg, Carl Skottsbergs gata 22B, 413 19 Göteborg, Sweden

[10] Department of Atmospheric and Oceanic Sciences, University of California Los Angeles, Los Angeles, CA 90095, USA

[*] To whom correspondence should be addressed. Email: ttreude@g.ucla.edu

Keywords: Sulfate reduction | Methanogenesis | Acetate | IODP 370 temperature limits | International Ocean Discovery Program

Study of potential anaerobic oxidation of methane (AOM) activity

One month after anoxic samples were shipped to the shore-based laboratory at UCLA and stored at 4ºC, samples were prepared for the study of potential activity of anaerobic oxidation of methane (AOM) with radioisotopes (^{14}C-CH_4). For this, approximately 5 mL of sediment from the innermost, undisturbed part of a WRC was placed into a 12 mL crimp vial to which 3 mL of seawater medium was added (see main text for recipe and procedures to prevent contamination during sample processing). Three replicate vials were prepared from each sample. Vials were crimp-sealed with non-toxic blue chlorobutyl stoppers (Bellco) and aluminum crimps. After sealing, the vial headspace was flushed with CH_4 for one minute and the sample was stored at room temperature in the dark for ~5 days until further processing and to let the sample liquid equilibrate with the methane headspace. After equilibration, the headspace was removed from all vials with a needle syringe by simultaneously replacing the headspace with methane-saturated sulfate reducer medium with a second needle syringe to fill the vial bubble-free. Prior to radiotracer addition, all vials were pre-incubated at their final incubation temperature (see below) for a couple of hours. To prevent formation of a gas headspace in the vial during heating and to allow expanding water to escape, vial stoppers were prepared with sterile needles that allowed gas and liquid to degas/drain. However, complete prevention of CH_4 headspace formation due to degassing was not possible. All samples therefore had a small (~ 0.5-1 ml) CH_4 headspace prior to radiotracer incubation. After initial heating, needles were removed from the stoppers and 10 µL of radiolabeled, gaseous (^{14}C) CH_4 containing up to 12 kBq of radioactivity was injected through the rubber stopper. Samples were shaken vigorously and incubated at temperatures within the in-situ range: 40°C for ≤ 360 mbsf, 60°C for 405-585 mbsf, 80°C for 604-775 mbsf and 95 °C for ≥ 816 mbsf). After 71-73 days of incubation, sample vials were opened, and the samples were quickly transferred into 50 ml glass crimp vials filled with 25 ml NaOH (2.5% w/w) and sealed with rubber stopper and aluminum crimp. Please note that during this transfer all ^{14}C-labeled and unlabeled CH_4 in the headspace was lost. Hence, only CH_4 that was dissolved in the sample was captured in the final sample vial. Medium controls (c_M; methane-saturated seawater medium, no sediment) were incubated at the four temperatures with radiotracer addition to check for non-biological reactions in the incubation medium. AOM samples were analyzed by gas chromatography, ^{14}C-CH_4 combustion, and ^{14}C-CO_2

acidification and trapping as described in detail previously[1]. Since the methane headspace was lost during transfer of the sample into the final sample vial, calculation of AOM rates was not possible. Instead, we present cpm (counts per minute) of $^{14}C-CO_2$ produced by AOM as compared to the medium controls. A minimum quantification limit (MQL) was calculated based on the average activity measured in the respective medium control (c_M) incubations plus 3 times standard deviation of the medium controls (Fig. S2).

Supplementary Figure Legends

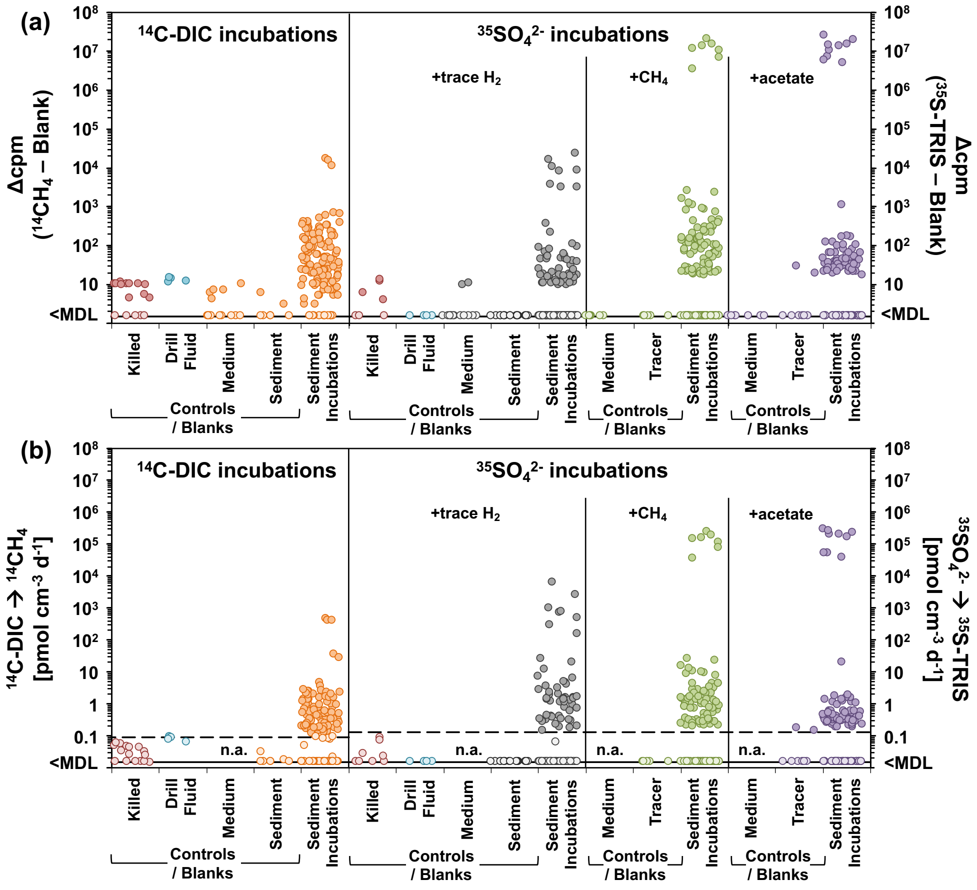

Figure S1 | Radiotracer turnover in incubation experiments and controls. Produced $^{14}CH_4$ in ^{14}C-DIC incubations and produced ^{35}S-TRIS in $^{35}S\text{-}SO_4^{2-}$ incubations are presented (a) in Δcpm, i.e. counts per minute radioactivity above the counter blank signal (see Table S1), or (b) sample volume-specific process rate (pmol per cm^3 sediment or drill fluid per day). The minimum detection limit (MDL) represents the average count rate measured in the respective counter blanks plus 3 times standard deviation. Dashed lines in (b) depict the minimum quantification limit (MQL) for methanogenesis and sulfate reduction, based on the average radioactivity measured in the killed control incubations plus 3 times standard deviation, which was 0.09 pmol CH_4 cm^{-3} d^{-1} and 0.13 pmol SO_4^{2-} cm^{-3} d^{-1}, respectively. n.a. = not available. Different incubations are represented by different colors, and incubations with activities <MDL or <MQL are represented by open circles.

For methodical details of all procedures, and definitions of blanks and controls see text. Source data as a Source Data file.

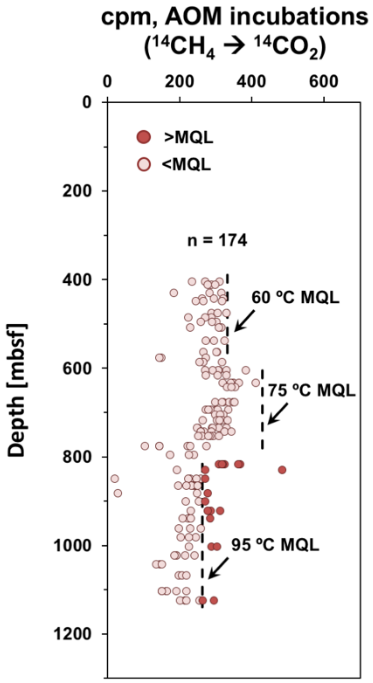

Figure S2 | ^{14}C-CO_2 production by anaerobic oxidation of methane (AOM). Produced ^{14}C-CO_2 is presented in counts per minute (cpm) radioactivity. Dashed lines depict the minimum quantification limit (MQL) determined from the medium control at the respective incubation temperatures. No killed control experiments were performed for AOM incubations, as γ-irradiated samples became available only after the incubation. Hence, the determination of the threshold for AOM activity was different from sulfate reduction and methanogenesis and therefore radiotracer turnover should not be directly compared. Note that ^{14}C-CO_2 builds up abiotically over time during ^{14}C-CH_4 incubations in water due to chemical decomposition of methane by radicals. This build up is insignificant in samples with medium to high AOM activity (e.g., surface sediments and methane seeps) but can interfere with ultra-low activity. In all incubations, dissolved methane was provided at approximately 100% saturation. For more details on the experimental setup, please refer to the methods. Source data as a Source Data file.

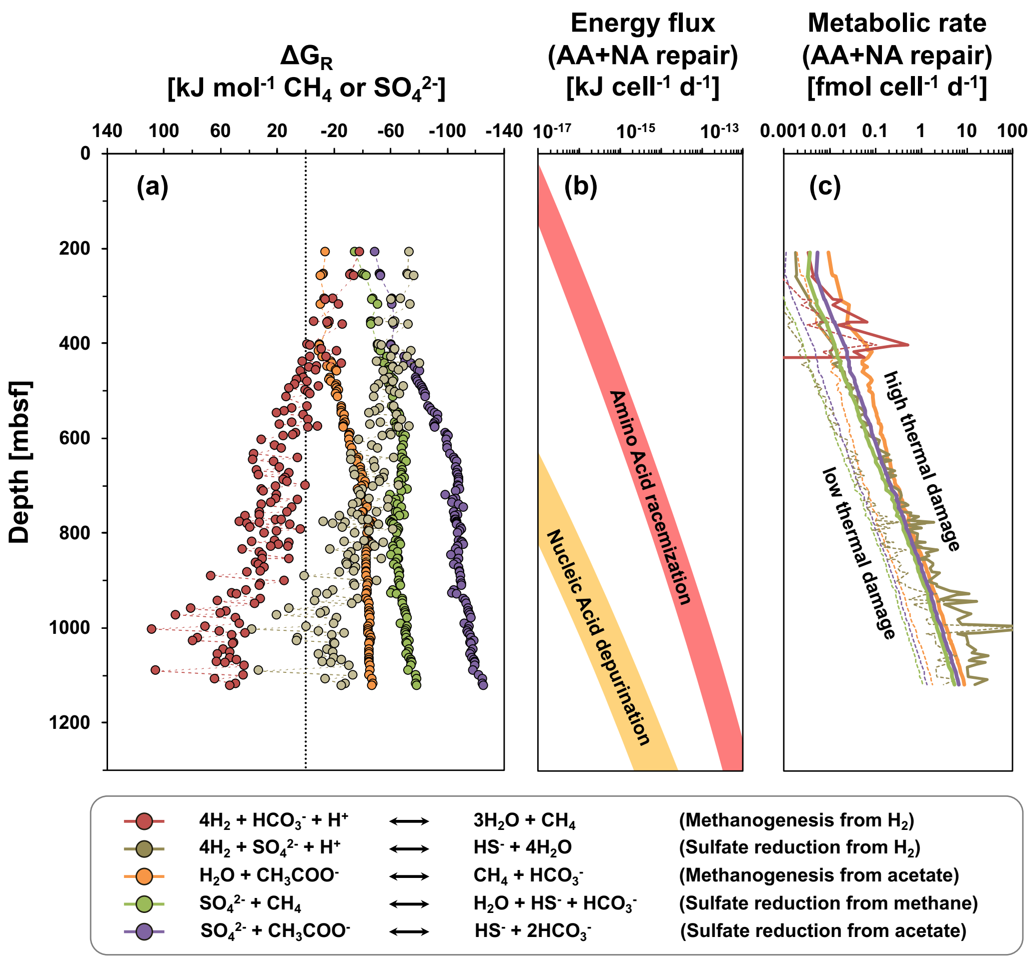

Figure S3 | Microbial energy yield and thermal damage in C0023 sediments. (a) Calculated Gibbs free energy yields of sulfate-dependent AOM, sulfate reduction from acetate, sulfate reduction from hydrogen, methanogenesis from acetate, and methanogenesis from hydrogen, energy flux, (b) cell-specific energetic cost of DNA depurination and racemization repair[2], and (c) metabolic rates necessary to account for lowest and highest estimates (dashed and solid lines, respectively) of thermal damage to DNA and amino acids**.** Source data as a Source Data file.

Supplementary Tables

Table S1. ^{35}S-TRIS and $^{14}CH_4$ activity in blank and control measurements (left column) that accompanied different incubation series (top row). For definitions of blanks and controls see text.

	^{35}S-SO_4^{2-} incubations [a]										^{14}C-DIC incubations [b]			
	+trace H_2				+CH_4		+Acetate		Killed controls		+trace H_2		Killed controls	
	(on board)		(shore-based)		(shore-based)		(shore-based)		(shore-based)		(on board)		(shore-based)	
Blank or Control	cpm	N	cpm	N	cpm	N	cpm	N	cpm	N	cpm	N	cpm	N
Counter	18.5 (±3.0)	6	13.0 (±1.0)	4	20.2 (±5.2)	5	20.2 (±5.2)	6	34.8 (±1.3)	5	18.9 (±0.9)	18	19.6 (±1.3)	7
Distillation	19.9 (±1.9)	21	17.8 (±4.9)	15	23.7 (±5.3)	30	24.2 (±6.1)	31	43.8 (±13.2)	8	n.d.		n.d.	
Tracer	n.d.		18.1 (±2.5)	7	26.2 (±7.6)	5	26.6 (±6.9)	7	n.d.		n.d.		n.d.	
Medium	18.8 (±2.8)	15	18.7 (±2.8)	14	22.7 (±5.1)	7	26.5 (±8.4)	8	42.3 (±12.6)	5	21.6 (±3.6)	14	n.d.	
Sediment	19.11 (±2.3)	9	n.d.		n.d.		n.d.		n.d.		20.0 (±2.6)	7	n.d.	
Drill Fluid	20.8 (±3.0)	4	n.d.		n.d.		n.d.		n.d.		32.0 (±1.8)	4	n.d.	
Killed	n.d.		n.d.		n.d.		n.d.		39.0 (±7.2)	7	n.d.		26.3 (±4.0)	16

[a] Radioactivity of ^{35}S-TRIS is presented as average cpm (±standard deviation)
[b] Radioactivity of $^{14}CH_4$ is presented as average cpm (±standard deviation)
cpm Counts per minute radioactivity
N Number of samples
n.d. Not determined

References

1. Treude, T., Krüger, M., Boetius, A. & Jørgensen, B. B. Environmental control on anaerobic oxidation of methane in the gassy sediments of Eckernförde Bay (German Baltic). *Limnol. Oceanogr.* **50**, 1771–1786 (2005).
2. Lever, M. A. *et al.* Life under extreme energy limitation: a synthesis of laboratory- and field-based investigations. *FEMS Microbiol. Rev.* **39**, 688–728 (2015).
